# Starch Modification with Molecular Transformation, Physicochemical Characteristics, and Industrial Usability: A State-of-the-Art Review

**DOI:** 10.3390/polym15132935

**Published:** 2023-07-03

**Authors:** Ruidi He, Songnan Li, Gongqi Zhao, Ligong Zhai, Peng Qin, Liping Yang

**Affiliations:** 1 School of Food Engineering, Anhui Science and Technology University, 9 Donghua Road, Fengyang 233100, China; ruidihe@163.com (R.H.);; 2 Joint International Research Laboratory of Agriculture and Agri-Product Safety of the Ministry of Education of China, Institutes of Agricultural Science and Technology Development, Yangzhou University, 48 Wenhui East Road, Yangzhou 225009, China; lsnyz2020@yzu.edu.cn

**Keywords:** modified starch, multi-functionality, physical treatment, chemical treatment, enzyme treatment, biological treatment

## Abstract

Starch is a readily available and abundant source of biological raw materials and is widely used in the food, medical, and textile industries. However, native starch with insufficient functionality limits its utilization in the above applications; therefore, it is modified through various physical, chemical, enzymatic, genetic and multiple modifications. This review summarized the relationship between structural changes and functional properties of starch subjected to different modified methods, including hydrothermal treatment, microwave, pre-gelatinization, ball milling, ultrasonication, radiation, high hydrostatic pressure, supercritical CO_2_, oxidation, etherification, esterification, acid hydrolysis, enzymatic modification, genetic modification, and their combined modifications. A better understanding of these features has the potential to lead to starch-based products with targeted structures and optimized properties for specific applications.

## 1. Introduction

Starch is primarily derived from plant carbohydrates and is a renewable biopolymer that is found in various plant tissues [1,2], such as the endosperm of cereals, thin-walled tissues of tubers, cotyledons of legume seeds [3], and the pulp of immature fruits [4]. Starch can be spherical, elliptical, polygonal, flattened, elongated, or kidney-shaped [5]. Natural starch consists of amylose and amylopectin [6], and it presents a complex semi-crystalline structure with a starch granule density of 1.5 g/mL. The density of the starch granules is higher than the density of fibers (density range: 1.2–1.4 g/mL) [7]. The molecular weight of amylopectin is significantly higher than that of amylose, with most samples containing between 15% and 30% of amylose [8].

Starch is a readily available and abundant source of biological raw materials and is widely used in the food, medical, and textile industries. However, as the application prospects of starch continue to improve, the limitations of using natural starch become more prominent. For instance, natural starch is insoluble in sluoat room temperature, is prone to retrogradation, and is susceptible to viscosity loss under low pH and high-temperature conditions. These properties significantly hinder the application prospects of starch [9]. Researchers have been focusing on methods to modify starch to improve the physicochemical characteristics of natural starch derived from different sources. Physical, chemical, biological, and combined modification methods can be used to address the problems associated with the use of natural starch. These modification methods have been reviewed in this paper.

## 2. Physical Modification

Physical modification technology is an economical and eco-friendly technology that can be used to modify starch properties. Physical modifications can usually be divided into thermal and non-thermal treatments [10]. Heat treatment and non-heat treatment refer to the functional properties of starch that can be tuned by controlling the temperature and moisture levels in the absence of foreign substances [11]. Currently, the physical-modification method has emerged as an important starch-modification method.

### 2.1. Thermal Treatment

#### 2.1.1. Hydrothermal Treatment

Hydrothermal treatment is a physical modification method that preserves the surface characteristics of starch granules and that maintains the starch structure better than other physical modification methods. The common methods include annealing (ANN), heat moisture treatment (HMT), and superheated steam (SS). ANN and HMT are executed above the glass transition temperature and below the gelation temperature of starch [12,13]. The execution of the SS method results in an increase in the temperature of starch above its saturation point as steam is released at a given pressure during the process. This method was superior to conventional hydrothermal modification methods, and the use of SS can save 50–80% of energy [14,15,16]. This can be attributed to the high-heat-transfer coefficient of superheated steam. The high-heat-transfer coefficient results in an increase in the mobility of starch molecules, promoting the process of modification. Additionally, the SS method was safer than the HMT method [17,18]. Adebowale et al. investigated the effect of ANN and HMT on the properties of millet starch and found that the modified starch contained <1% of broken granules [19]. The solubility tended to increase with increasing humidity, and the swelling capacity of the materials subjected to these conditions was lower than that of natural finger millet starch. The pasting temperature of the starch samples subjected to ANN and HMT increased while the pasting viscosity decreased. Dutta et al. and Shi et al. found contrary results, who reported that the swelling capacity and solubility of potato starch subjected to ANN and HMT methods were lower than those of natural potato starch [13,20]. This may be attributed to the depolymerization and damage of starch granules. The strong interactions between amylose and amylopectin molecules prevented their leaching from the granules [16,21,22]. The results reported by Xiang et al. confirmed the results reported by Dutta et al. and Shi et al. [13,20,23]. They pointed out that the ANN-based modification of starch, realized at the hydrothermal treatment temperature, promoted the rearrangement of starch molecular chains, resulting in the formation of a perfect crystal structure.

The results reported by Ma et al. agreed well with the results reported by Dutta et al., Shi et al. and Xiang et al. [13,20,22,23]. They reported that the swelling power, solubility, leached amylose content, viscosity, and ΔH (enthalpy change) of starch subjected to conditions of hot steam treatment were lower than those of natural starch. Liu et al. demonstrated that starch from different sources exhibited different gel states after SS treatment [16]. This variability could be attributed to the nature of starch, the heating method used, and the processing conditions. Therefore, modified starch samples can be selected based on specific requirements, and those with similar properties can be used interchangeably [24]. In conclusion, the SS method can reduce the swelling ability and improve the starch gel stability. Due to its high efficiency and low loss characteristics, this method will be a promising method for starch modification and can be widely applied in the future.

#### 2.1.2. Microwave

Microwave treatment involves the use of electromagnetic radiation at frequencies between 300 MHz and 300 GHz [25]. These radiations affect daily life, and the microwave treatment method is an efficient and green method that can be used to modify starch. The electric field of microwave radiation vibrates at high frequency, leading to the continuous orientation of polar and ionizable molecules. The altered molecules rub together and collapse with surrounding molecules through electromagnetic induction and then generate heat [26]. Li et al. studied the physicochemical properties and in vitro digestibility of sorghum starch under conditions of microwave treatment [27]. The results revealed that the surface of the three sorghum starch granules was rough, and the pasting temperature and pasting time increased post microwave treatment. The slowly digestible starch and resistant starch contents increased significantly when the samples were subjected to conditions of microwave treatment, and the process did not alter the chemical functional groups of the starch samples. Wang et al. compared the effects exerted by ultrasonic and microwave treatment methods on the structural and thermal properties of common corn and potato starch samples [28]. They found that microwaves and ultrasound helped to reduce the total amylose starch content in starch. This may be due to the disruption of the surface of starch granules and the double helix of starch. The effect of modification was influenced by the source of starch. The ease of operation, “green” with nature, and sustainability of microwave technology make it a promising starch-modification technology. In addition, microwave technology can increase the resistant starch content of native starch, providing a new method for the development of low GI products.

#### 2.1.3. Pregelatinization

Pregelatinized starch has a special structure, such as hydrogen bond breaking porosity, where the crystal zone in the pregelatinized starch particles is completely or partially destroyed and has properties such as cold-water solubility and high viscosity compared to natural starch. It can reduce the processing time and heat loss in later applications, thus shortening and simplifying the process. For instance, pregelatinization is used for thickeners in many instant food products. These kinds of products require only the addition of hot water with stirring before serving, such as baby food, instant soups and instant deserts. Pregelatinized starch is a kind of starch modified by physical modification. It can significantly change the viscosity, water-binding ability, gel structure, and other properties of starch. Adding pregelatinized starch to natural starch, or the direct replacement of natural starch with pregelatinized starch, can shorten and simplify the production process while improving the quality of the final product [29]. The concept of “convenience living” has attracted the attention of consumers, and this has resulted in an increase in the production of instant food items that can be consumed by heating. Therefore, methods of processing starch-based pregelatinized foods are being extensively studied. Pregelatinized starch can be oven dried, spray dried, drum dried, extruded dried, and freeze dried. Among these methods, oven drying is the oldest known drying method. The uniformity of oven drying temperature is difficult to control. The disadvantages of using this method have hindered the application prospects of the method, and the use of this drying method is being gradually restricted.

#### 2.1.4. Spray Drying

Spray drying is a method of modified starch that combines several drying steps into one, resulting in reduced drying time. This method can be efficiently used to realize the mass production of samples. Tay et al. introduced a low-temperature spray-drying process that yielded glutinous rice starch in high yields [30]. The product was characterized by low moisture content and uniform particle size. The crystallinity and pasting properties of the spray-dried glutinous rice starch were retained during the treatment process. In addition, in the spray-drying method, different pretreatment conditions have different effects on the physicochemical properties of starch. Santos et al. pasted and spray-dried natural cassava starch at different preheating temperatures [29]. They showed that spray-dried tapioca starch (starch concentration of 25%, preheating temperature of 52 °C, processing heating time of 10 min) shows a low change in gelation enthalpy and a high gelatinization temperature and gelatinization degree, which make it suitable for industrial use as a partially gelatinized starch. They also varied the starch concentrations and heating times to understand the effects of different physical conditions on the properties of starch and reported that the preheating temperature and heating time had a significant effect on cold viscosity. These results can help in the production of fast-brewing powders and gels that can be used to develop convenience foods. These authors have published several papers on the process followed for the modification of starch. They reported that the spray-drying method affected the physicochemical properties (solubility, swelling, and pasting properties) of starch. Starch solubility was not significantly affected, and the effect of the treatment conditions on the viscosity of starch was low [29,31,32,33]. In spray drying, the preheating temperature and starch concentration affected the solubility, swelling, crystallinity and pasting temperature of the starch. Although the spray-drying method has conventionally been used for the production of pregelatinized starch, the high cost of the production process, complex influence factors and difficulties of achieving consistent quality limit the application of this method.

#### 2.1.5. Drum Drying

Drum drying involves two stages (pasting and drying) and can be accomplished using single-drum or double-drum dryers [34]. This method was considered more convenient and cost-effective than spray drying and extrusion drying. Chittrakorn et al. investigated the process of preparation of pregelatinized sweet potato starch at different temperatures (110, 120, and 130 °C) following the process of drum drying [35]. The results indicated that an increase in the pregelatinization temperature significantly increased the water absorption index of pregelatinized starch. Furthermore, the water solubility index of this sample was lower than that of natural sweet potato flour. The addition of an appropriate amount of pregelatinized sweet potato flour to wheat flour resulted in an improvement in cake quality. In another report, Obadi et al. examined the influence of pregelatinized starch (treated under different conditions) on dough characteristics and soba noodle quality and found that the dough tensile strength reached its maximum with the addition of blast-dried tapioca starch [36]. As a whole, the viscosity and water absorption index of drum-dried starch was higher than those of the spray-dried and extruded starch samples. Further research should focus on expending the application prospects of drum-dried modified starch.

#### 2.1.6. Extrusion

Starch extrusion technology is a thermo-mechanical process that breaks the bonds in starch, resulting in the pasting, melting, and degradation of starch. This technology involves the execution of several unit operational steps, such as mixing, cooking, kneading, shearing, shaping, and forming [37]. Extrusion is a cost-effective modification method that can be used to achieve high productivity. Various combinations of parameters can be chosen to execute this versatile method to obtain the desired end product [38,39]. The final products are characterized by low density and high expansion ratio [40].

Gandhi et al. investigated the effect of twin-screw extrusion on the properties and quality attributes of corn and potato starch [39]. The water absorption index, water solubility index, degree of starch pasting achieved, and in vitro digestibility of the extruded starch samples were significantly higher than those of the untreated starch samples. The polysaccharide chain molecules and intermolecular hydrogen bonds in starch molecules break during extrusion. This results in the transformation of the samples from their native state to the crushed and pasted states [41]. This facilitates the water absorption of starch molecules and promotes the swelling and increase in the water absorption index. The water solubility index increase can be explained by the increase in the extrusion temperature, the decrease in molecular weight due to high mechanical shear [42], and gelatinization [43].

The results differ depending on the raw material used, extrusion parameters, and extrusion conditions. Qi et al. studied the extrusion process of pea starch (moisture content: 25–55%) using a twin-screw extruder (aspect ratio: 26) [44]. The lowest relative content of resistant starch was observed when the moisture content was 40%, the screw speed was kept at 180 r/min and the shear temperature was 70 °C. When the moisture content was 25%, screw speed kept at 140 r/min and shear temperature at 70 °C, the relative content of slowly digested starch was the lowest. Li et al. extracted starch from pineapple honey seeds and used a double-screw extruder to modify the precipitated powder by controlling the extrusion operation parameters [45]. The results were in agreement with Qi et al. that the fine structure of pineapple honey seed starch was significantly influenced by the extrusion operation parameters. Ali et al. found that the high temperature and shear force caused a disruption in the hydrogen bonds and molecular structure of potato starch, resulting in reduced stability and lower molecular weight [46]. The high temperature and shear force also led to an increased gelatinization degree and tighter arrangement of starch molecules [47]. Therefore, the setting of the parameters during the extrusion process determined the properties of the starch.

Improved extrusion cooking technology (IECT) improves the performance of the extrudate. A low amount of energy is consumed during the process, and IECT is a promising and improved method that can be used to treat and process starch. Cheng et al. followed the IECT process for the extrusion of buckwheat flour, and the results revealed that the IECT-treated buckwheat flour samples retained favorable functional components and physical properties [47]. However, the extrusion process has much room for development, and its application prospects can be improved by conducting in-depth studies on the physicochemical properties of the samples.

In conclusion, the parameters of the pregelatinization method are universal, and pregelatinized starch has excellent pasting properties, which can be used in a wide range of applications. Each pregelatinization treatment has advantages and disadvantages, therefore, it is necessary to further optimize the pregelatinization method to meet the needs of different food industries.

### 2.2. Non-Thermal Treatment

#### 2.2.1. Ultrasonication

**Figure 1:**
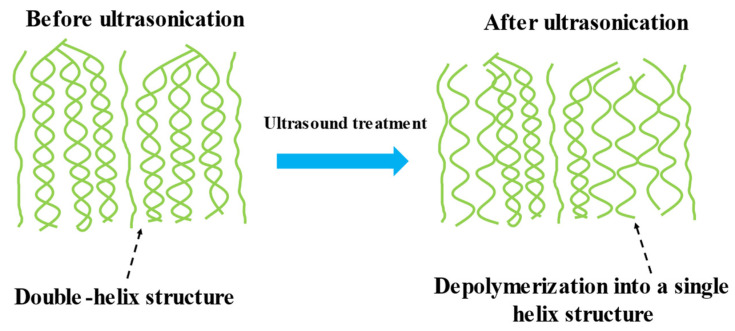
Schematic diagram of starch modification with ultrasound [51].

Ultrasonic modification is a processing technique that results in the physical depolymerization of starch. Ultrasound helps to improve the physicochemical properties of starch. The advantages of the process lie in the use of small amounts of chemicals and the consumption of less amount of time [48]. Satmalawati et al. varied the amplitudes and treatment durations and used eight combinations of amplitudes and treatment times to treat starch [49]. They reported that ultrasound treatment degraded starch polymers, while resulting in the generation of amorphous starch. Thus, modified starch had a higher content of amylose, low viscosity, and increased solubility of the samples. The results agree well with the results reported by Mohammad Amini et al. and Rahaman et al. [48,50]. Ultrasonic modification is an ideal modification method because it is a green method, and the modification parameters can be adjusted without restrictions. This method can be used to obtain modified starch with the desired properties. However, the effect of ultrasonic modification was unstable, and the experimental results were difficult to reproduce. In the future, we need to explore the principle of ultrasonic modification in depth to obtain the target properties of starch. The changes in the properties of starch after ultrasonic modification are shown in Figure 1.

#### 2.2.2. Radiation

**Figure 2:**
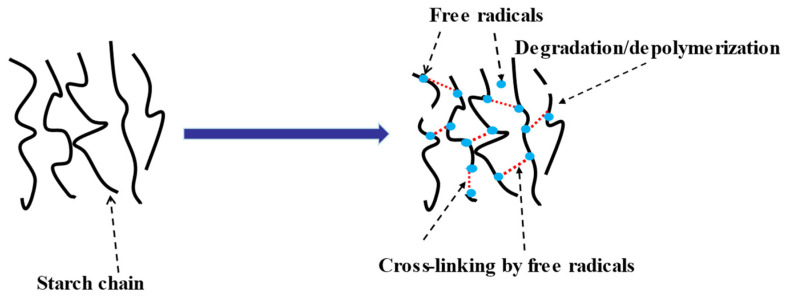
Schematic diagram of the effect of radiation on starch [55].

Radiation technology can be potentially used to improve the solubility and enzymatic digestibility of starch, and thus, it can be widely used in the food industry [52]. Radiation-based modification techniques are safe and effective tools that can be used to modify starch. The effect of radiation on starch is shown in Figure 2. Irradiation significantly increases the water solubility of irradiated starch and significantly reduces the viscosity of the samples [52,53]. Liang et al. treated starch under conditions of varying irradiation doses (6, 12, and 24 kGy) [54]. They also varied the frequencies (1, 2, 4, and 8 times) of the electron beam used for irradiation. The results revealed that an increase in dose resulted in an increase in the extent of starch degradation, a decrease in molecular weight and viscosity, and an increase in starch solubility and swelling capacity of the samples. The use of multiple low-dose irradiations helped improve starch properties and reduce energy consumption. The physicochemical properties of starch recorded under conditions of irradiation can complement some of the existing properties of starch, thereby improving the application prospects of starch.

#### 2.2.3. Ball Milling

**Figure 3:**
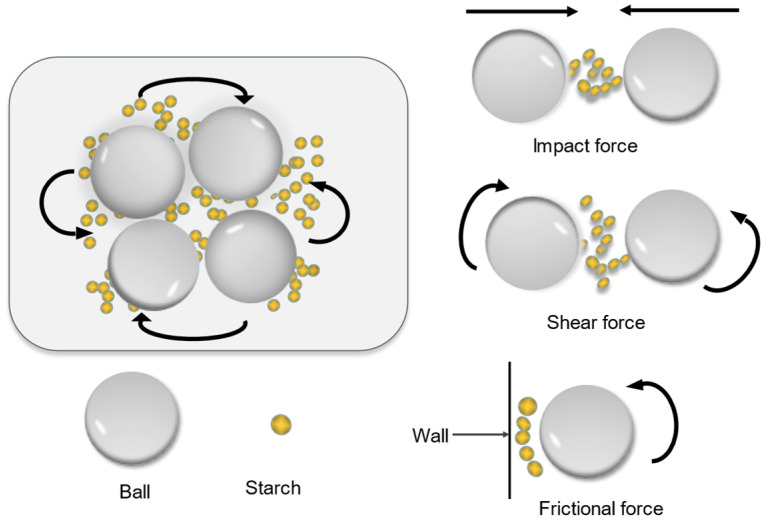
The interaction mechanism of ball milling [62].

Ball milling modification involves the exploitation of friction, impact, shear, or other mechanical forces to modify the structure and properties of starch granules (Figure 3) [56]. Ball milling modification can be categorized into dry ball milling and wet ball milling methods. Wet ball milling is an energy-efficient method that can dissipate heat effectively and produce samples with small particles. This method was more efficient than the dry ball milling method [57]. The effect of ball milling treatment primarily depends on the starch source and grinding conditions (density of the grinding material, ball size, grinding speed, grinding time, ball-to-powder ratio, etc.). Huang et al. investigated the effect of ball milling on the physicochemical properties of cassava starch and corn starch and reported that the ball milling process destroyed the crystal structure of starch [58]. It was observed that the pasting temperature of starch decreased with an increase in the grinding time, the cold-water solubility increased with the breakage of hydrogen bonds, and the cold-water solubility of cassava starch was higher than that of corn starch. Ahmad et al. used a planetary ball mill (600 rpm, 5 h, 5 zirconium balls with a diameter of 8 cm) to grind water caltrop and horseshoe starch [59]. The results indicated that the water absorption capacity and thermal stability of the modified starch were better than those of natural starch, and this result was consistent with the results reported by Huang et al. and Juarez-Arellano et al. [58,60]. Han et al. investigated the effect of treatment times (0, 10, 20, 30, 40, 50, and 60 min) on the granule structure and physicochemical properties of different starch samples (wheat starch, type A and type B starch) [61]. Scanning electron microscopy (SEM) technique was used for sample analysis, and the results revealed that the degree of starch granule fragmentation realized increased with an increase in the ball milling time. In contrast, partial re-agglomeration of wheat starch granules and type B starch was realized when the samples were ball milled for a prolonged time (30, 40, or 60 min). The re-agglomeration properties of starch significantly affected the digestibility of the samples. Soe et al. investigated the effect of milling time on the properties of glutinous rice starch [56]. The results revealed that the solubility of starch significantly increased with an increase in the ball milling time, and the starch swelling capacity reached its maximum at 15 min. Moreover, the longer the ball milling time, the lower the swelling capacity, and this can be attributed to the formation of different gelling fragments and soluble substances during the process of ball milling. Ball milling and grinding are simple processes that are environmentally friendly, and the resulting product is characterized by high solubility and good dispersibility. Thus, it can be inferred that the ball milling method is a promising physical modification method.

#### 2.2.4. High Hydrostatic Pressure (HHP)

**Figure 4:**
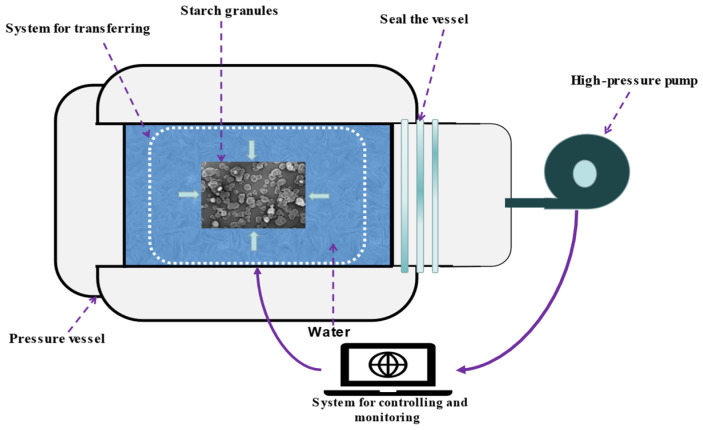
Schematic representation of the action of HHP processing [68].

The HHP-based treatment method is a new, non-thermal, and environmentally friendly treatment technique that is easy to control [63]. This method can be used for physical modification. The mechanism of HHP action on starch is shown in Figure 4. Rahman et al. studied the effect of different parameters of HHP (HHP100, 300 and 500 MPa, 15 and 30 min at 52 °C) on the physicochemical, thermal, and structural properties of different varieties of starch samples [64]. The results revealed that the amylose and damaged starch contents of the starch samples increased following HHP treatment. However, the solubility and swelling capacity of the samples decreased, and significant differences in thermal properties between the different varieties of starch samples were observed. This can be potentially attributed to the different degrees of HHP-induced pasting [65]. However, the high hydrostatic pressure ensures that the forces acting on the product are uniform and that it is energy efficient and retains the product’s nutrients well. During food processing, starch physically interacts with fatty acids, proteins, polyphenols and other substances to form starch complexes. These complexes are useful in food technology, as they influence the texture, physicochemical properties and quality of foods. For example, the incorporation of a high amylose starch, fatty acid complex into surimi resulted in a more compact network structure of surimi gel, which improved the quality of surimi products [66]. Guo et al. prepared lotus seed starch, fatty acid complexes using high hydrostatic pressure, analyzed their physicochemical properties and found that the complexes presented a compact structure, higher crystallinity and higher thermal stability. In addition, it was demonstrated that the crystalline properties of the complexes were related to pressure and fatty acid chain length [67]. The application of HHP technology in preparation of starch–fatty acids complexes further expands the range of applications of this modification method.

#### 2.2.5. Supercritical CO_2_

**Figure 5:**
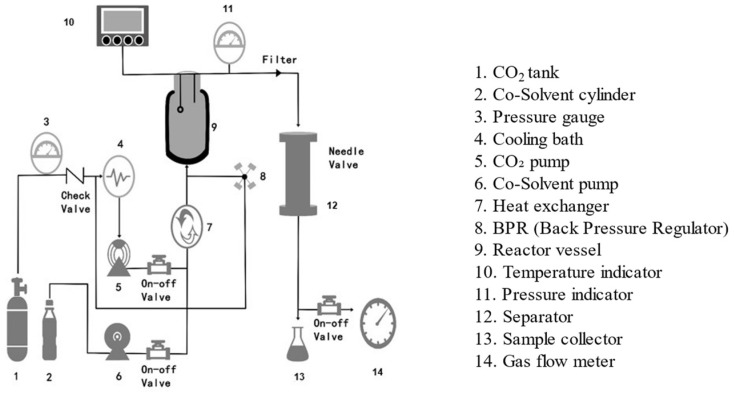
Schematic diagram of supercritical CO_2_ unit [72].

A supercritical fluid is a compound that is formed above critical pressure and temperature and is a green solvent that is inexpensive, safe, non-toxic, and environmentally friendly [69]. CO_2_, as a solvent in supercritical fluids, has an easy-to-control critical value, is convenient to use, and is characterized by a much lower critical temperature than other commonly used solvents. This fluid exhibits both gas and liquid properties, such as low viscosity, high diffusion coefficient, and good dissolution properties [70]. Villegas et al. studied the morphology and size-distribution properties of starch granules [71]. The results were recorded before and after contact with supercritical CO_2_. They also studied the solubility and volume expansion properties of the starch samples in supercritical CO_2_. Analysis of the SEM images revealed that the average diameter of the treated starch granules was higher than the average diameter of untreated starch granules when the samples were treated with supercritical CO_2_. This resulted in swelling of the starch granules. This may be due to the absorption of CO_2_ by starch. However, the extent of swelling realized in this case was negligible compared to the extent of swelling realized following other modification methods. The authors pointed out that the introduction of different functional groups exerted varying degrees of effects on the physicochemical properties of modified starch. Moreover, the supercritical fluid-modification approach can be combined with physical, chemical, and biological methods to obtain starch samples with the target properties. Figure 5 shows the schematic diagram of the supercritical CO_2_ unit. Table 1 summarizes the physical modification methods for the various starch sources that have been reported above.

## 3. Chemical Modification

Chemical modification involves the introduction of new functional groups into the polymer molecules. This results in significant changes in the physicochemical properties of starch molecules [73,74]. Starch contains α-1,4 glycosidic and α-1,6 glycosidic bonds, and the skeletal structure contains a reduced aldehyde end. Therefore, starch was prone to aldehyde- and base-induced depolymerization and glycosidic rearrangement [75]. Chemical modification methods can be divided into four categories: oxidation, esterification, etherification, and acid hydrolysis. Suitable treatment methods can be chosen based on the desired properties [76]. Food-grade and chemically modified starch must comply with relevant regulations, and the disposal of chemical waste generated during the treatment process poses a significant challenge. However, there is still a lack of research on the potential long-term effects of consuming chemically modified starch on human health. 

### 3.1. Oxidation

The oxidation of modified starch results in the conversion of hydroxyl groups to carbonyl and carboxyl groups, resulting in the depolymerization of amylose and amylopectin [77]. The chemical reagents widely used for starch oxidation are hydrogen peroxide, hypochlorite, periodate, dichromate, and ozone [78]. Oxidation modification results in improved gel texture, stability, and film adhesion [79]. However, chemical waste generated during starch modification needs to be treated appropriately after disposal. Additional concerns include low yields of modified starch, consumption of large amounts of wastewater and time, and the uncertainty about the safety of chemically modified starch.

Tung et al. studied the oxidation of starch extracted from pineapple honey seed [80]. They used hydrogen peroxide for oxidation and analyzed the starch samples using SEM, X-ray diffraction (XRD), and Fourier transform infrared spectroscopy (FTIR) techniques. The results revealed that starch underwent oxidation in the amorphous region, and rough and microporous granules were formed post oxidation. However, the safety of using this oxidized starch in the food industry was not reported.

The electrolysis technique can potentially address most of the problems associated with chemical modification. Food-grade chemicals are used in small amounts during electrolysis. Thus, these can be easily removed from the final products. Castanha et al. used tapioca starch as the raw material [81]. They oxidized the raw material in an electrolytic cell using sodium chloride as the electrolyte. The results revealed that the number of carbonyl and carboxyl groups in oxidized starch increased rapidly with an increase in the NaCl content, and the pores on the surface of the modified starch granules (observed using the SEM technique) increased in size under these conditions. The solubility of this sample was higher than the solubility of natural starch. This can be attributed to starch being primarily oxidized in the amorphous region. The starch molecules break down into smaller molecules, forming a network that readily absorbs water. This increases the solubility of the molecules [82]. This result agrees with the results reported by Tung et al [80].

**Figure 6:**
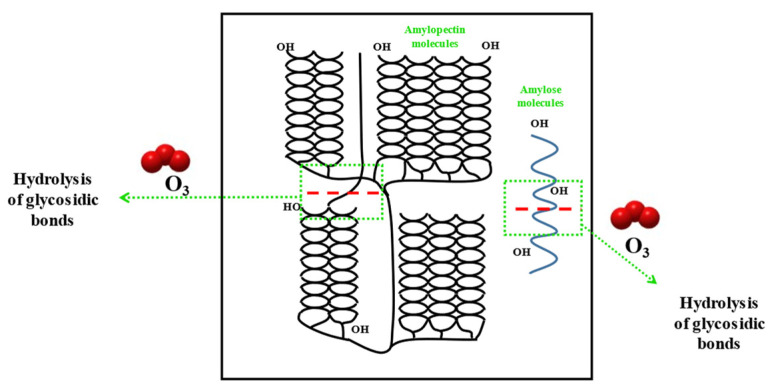
Mechanism of ozone action in starch molecules [87].

Ozone is a robust and environmentally friendly oxidant. It is a stronger oxidizing agent than sodium hypochlorite and hydrogen peroxide [83]. Ozone can be readily degraded to form oxygen and is not harmful to the environment. Moreover, it does not leave any residue in the final product [79]. The mechanism of action of ozone in the starch molecule is shown in Figure 6. Castanha et al. studied ozone-oxidized potato starch and reported that, unlike natural starch, the ozone-oxidized starch sample could be fully pasted at low temperatures [81]. It was also observed that the ozone-oxidized sample dissolved faster than the other samples, and the amount of water retained by the pasted samples was higher than that retained by native starch. This resulted in an increase in the apparent viscosity and gel strength of the samples. The results reveal that ozone treatment was better than other chemical treatment methods, as it is a safe and green method that can be used to produce starch with the desired properties. Hu et al. further confirmed the feasibility of producing ozone-modified starch and discussed the role of time in the field of ozone treatment of buckwheat starch [84]. The results revealed that aldehyde groups generated during oxidation during the preparation of starch paste in boiling water, at the ozone treatment times of 2.5 and 7.5 min, cross-linked with the hydroxyl groups of starch in the amorphous region. The amorphous layer of starch was further oxidized and degraded at ozone treatment times of 15 and 20 min. The ozone treatment method can be used to modify the structure and physicochemical properties of natural buckwheat starch. Thus, it can be potentially used as an ingredient in conventional products such as instant soup and meal replacement shakes. It can also be used in the field of 3D printing of food [85].

Ozone treatment is an efficient and widely used technology. A correlation analysis was conducted by Castanha et al. for the ozone treatment of cassava, corn, and potato starch samples [86]. The ozone-treated starch results in a product with high industrial relevance and low consistency, with higher strength after cooling and storage.

### 3.2. Etherification

**Figure 7:**
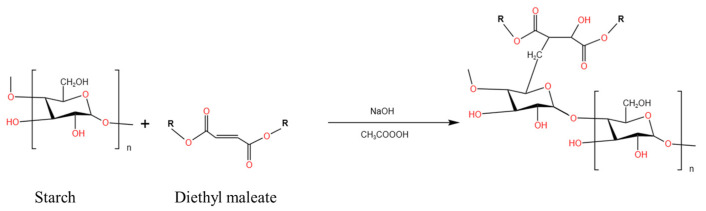
Mechanism of starch modified by etherification reaction [97].

Etherification is a modified method that, by introducing the lipophilic functional groups, is used to improve the water resistance of starch [88]. The mechanism of the etherification reaction is shown in Figure 7. Hydroxypropylation, hydroxyethylation, and carboxymethylation are the three etherification methods used to prepare starch-based ethers. Four types of modified products are usually obtained post etherification: nonionic, cationic, anionic, and amphoteric. Hydroxypropyl starch is the most common type of etherified starch, and it is produced using propylene oxide [89]. This paper focuses on reviewing low-substitution modified starch samples as the production of highly substituted starch samples involves the use of hazardous solvents.

Wang et al. used 3–9% propylene oxide to hydroxypropylate normal wheat starch, glutinous wheat starch [90], and glutinous corn starch and established the relationship between molar substitution and the degree of aging. Their results revealed that hydroxypropylation significantly affected the thermal properties of normal wheat starch, glutinous wheat starch, and glutinous corn starch. They reported that (1) the pasting and peak temperatures decreased with an increase in the hydroxypropyl content, (2) three hydroxypropylated starch samples were characterized by different degrees of swelling, and (3) the hydroxypropylated starch samples were difficult to age and degrade. Hydroxypropylated starch is commonly found in corn [91], amaranth [92], beans [89], rice [93], cassava [94,95], canna [96], etc. Although etherified starches can improve the sensory quality and overall properties of the final product, they are less frequently used in food products because it is difficult to guarantee the safety of etherifying agents added during the modification process. However, because of the high stability of etherified starches with low substitution, they can also be used as thickeners and stabilizers in food industries.

### 3.3. Esterification

**Figure 8:**
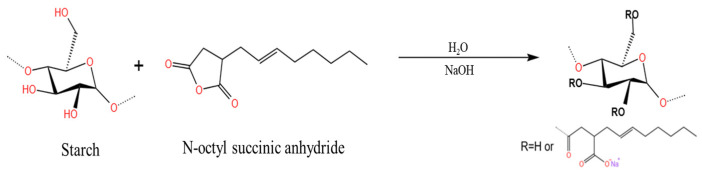
Mechanism of OSA-modified starch [108].

Esterification reactions can be conducted to effectively modify the structure of starch granules to improve the application prospects of starch. The skeletal structure of starch bears a large number of hydroxyl groups. Hence, starch can be esterified with acids and their derivatives [98]. Starch esters can be divided into inorganic and organic acid esters depending on the type of acid used for esterification [99]. According to the United States’ official regulations, esterified starch can be used in food products. The safety guidelines state that food items should not contain more than 3.0% octenyl succinic anhydrate (OSA)-modified starch [100,101,102]. OSA-modified starch is prepared by reacting starch with OSA, and it is one of the most important amphiphilic derivatives [100]. The reaction involves the introduction of a hydrophobic anhydride group into the starch chain to enhance the amphiphilic nature of the starch [103]. The method followed for OSA modification is the most widely used method of starch esterification because it does not require the use of toxic or hazardous organic solvents. Moreover, the desired results can be obtained under easily controllable experimental conditions [104]. The mechanism of OSA modification is shown in Figure 8. Zhang et al. investigated the effect of different degrees of substitution on the physicochemical properties of starch [105]. They used amylose as the raw material and esterified the samples with OSA at room temperature at a pH of 8.5. The results revealed that the form of the starch samples and the particle size did not change significantly post OSA modification. However, the relative crystallinity and pasting temperature of starch decreased, and the pasting viscosity, swelling capacity, and emulsion stability of the sample increased under these conditions. This result was consistent with the results reported by Zainal Abiddin et al. [106]. It was also observed that the physicochemical properties of OSA-modified starch varied based on the degree of substitution [107]. The disadvantage of OSA is its low solubility in water [107], but the improvement in swelling capacity, pasting viscosity, emulsion stability and shear resistance after OSA modification greatly expanded the application of OSA-modified starches in emulsions.

### 3.4. Acid Hydrolysis

**Figure 9:**
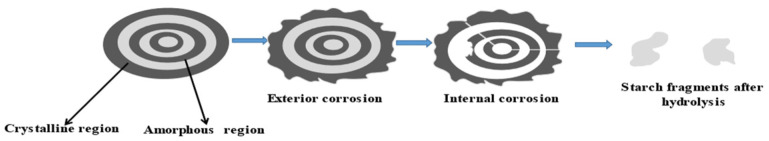
Acid hydrolysis process of granular potato starch [117].

A concentrated slurry is treated with acid at a temperature below the starch pasting temperature during acid hydrolysis. The method is also referred to as acid dilution [109]. Usually, starch is treated with inorganic acids (hydrochloric or sulfuric acid) during the process of acid hydrolysis [110,111]. The acid solution is neutralized with an alkali when the desired degree of hydrolysis is attained. Following this, the solution is washed [112]. Acid hydrolysis can significantly alter the structural and functional properties of starch. The process of starch acid hydrolysis is shown in Figure 9. In 1874, Nägeli produced the first hot-water-soluble starch following the process of sulfuric acid treatment. Lintner was the first to hydrolyze potato starch at room temperature using 7.5% (*w*/*v*) hydrochloric acid [113]. The modified starch was soluble in hot water, and the solution appeared colorless.

Abdorreza et al. studied the effect of acid hydrolysis on the physicochemical and rheological properties of sago starch [114]. It was observed that the solubility increased significantly after 12 h of acid treatment, and it was noted that more than 10 g of sago starch could be dissolved per 100 g of water. Mendez-Montealvo et al. used 7.5% hydrochloric acid for the hydrolysis of non-traditional plantain starch [115]. Hydrolysis was carried out over a period of 3–15 days. Acid hydrolysis resulted in damage of the amorphous as well as crystalline regions of sago starch. It was also observed that the solubility of the starch sample was significantly high on days 9 and 15, and this could be potentially attributed to the acid degradation of the samples at the level of the double helix structure.

Acid hydrolysis can impart specific properties to starch if the pH, hydrolysis time, and molecular weight of starches are controlled [116]. Although this is a simple and inexpensive modification method, few studies have been conducted to address the issues associated with low yields and the formation of undesirable byproducts. Table 2 summarizes the above reported chemical modification methods for various starch sources.

## 4. Enzymatic Modification

Enzyme modification technology can be used to effectively control the specific properties of starch. The use of biological modification methods helps to reduce the yields of byproducts. High specificity for hydrolysis products and high yields of target products can be achieved using these techniques [118,119]. Table 3 summarizes the enzyme modification methods for the various starch sources described in the text. Enzymatic modification is a green method that is closely related to the concept of world development. The enzymes usually used are α-1,4-glucanases and α-1,6-glucanases. It has been observed that a single enzyme does not significantly affect the properties of starch. Hence, a combination of multiple enzymes has been used by researchers to obtain the desired results. The biological enzyme modification method can be used to produce porous starch, slow-digesting starch, and resistant starch [120]. Enzymes can erode the entire granule surface or its parts (external erosion) or digest channels from selected points on the granule surface toward the center of the granule (internal erosion) [121]. The enzyme type, enzyme concentration, reaction time, and starch source play an important role in the physicochemical properties of the starch-based final product. Lopez-Ochoa et al. analyzed the paste properties and thermal properties of tapioca starch using pullulanase and amyloglucosidase [122], and their results revealed that the viscosity of the drinks prepared from pullulanase-modified starch was higher than that of the amyloglucosidase-modified starch. Compared with pullulanase-modified starch, a higher degree of long-term retrogradation was recorded for amyloglucosidase-modified starch [123]. Han et al. revealed that the desired final product could be prepared by controlling the type of enzyme, enzyme activity, and enzyme concentration [124]. Cornejo et al. investigated the effect of the degree of enzyme hydrolysis (0%, 30%, and 50%) on the properties of different varieties of cassava starch [125]. Structural analysis revealed that a progressive increase in the degree of enzyme hydrolysis resulted in progressive structural damage to cassava starch. Enzyme hydrolysis increased the water-holding capacity and water-binding capacity of the starch. Analysis of the rheological properties of starch revealed that the response of starch varieties to enzymatic hydrolysis was influenced by the degree of enzyme hydrolysis. However, the time-consuming nature of the enzyme-based treatment method hinders its application prospects, and researchers have conducted relevant studies to address this problem. Wang et al. introduced the use of ultrasound-based treatment methods in the glycolytic enzyme pretreatment, starch pretreatment, and mixed reaction system-based treatment methods to improve the enzyme hydrolysis efficiency of starch [126]. The enzymatic reaction conducted following ultrasonic pretreatment improved the degree of starch enzymatic hydrolysis (effective temperature: <65 °C), and the degree of enzymolysis hydrolysis varied with the experimental steps associated with ultrasonic treatment. In conclusion, the enzyme modification method is a promising and attractive method for starch modification.

## 5. Genetic Modification

Genetic modification of starch not only helps in understanding the mechanisms associated with the biosynthesis of starch but also presents a platform to expand the application prospects of starch. Biogenetic mutations impart starch with unique physicochemical properties. Wang et al. performed targeted mutagenesis with IbGBSSI and IbSBEII (genes associated with biosynthetic pathways in sweet potatoes) using CRISPR/Cas9 technology and found that amylose content in modified starch was higher than that in natural sweet potatoes [127]. Luo et al. successfully produced high amylose tapioca starch by CRISPR/cas9-mediated starch branching enzyme 2 (SBE2) mutation. It was found that the resistant starch and amylose starch content of genetically modified cassava starch increased significantly, and the starch viscosity changed with increasing pasting temperature and peak time [128]. Products with high amylose starch content contained higher levels of resistant starch, which was beneficial for human health [129]. Sun et al. successfully produced high amylose rice by CRISPR/Cas9-mediated targeted mutagenesis of SBEIIb and found that high amylose starch cereal products could provide new options for people with chronic diseases such as diabetes and obesity [130]. Fu et al. successfully obtained four waxy rice starches by CRISPR/Cas9 gene-editing technology and found that the above four mutants had different physicochemical properties, which provided a reference for obtaining rice with target properties in the future [131]. Waxy starch was known as low-amylose starch or amylose-free starch and offers special advantages in starch-based food processing. For instance, waxy rice was suitable for sauces, gravies and baby food, as it had higher viscosity and was easier to digest [132]. The development of the field of genetic engineering can contribute to the development of novel starch samples that can contribute to the future of food and agriculture. Table 4 summarizes the genetic modification methods for the various starch sources described in the text.

## 6. Dual Modifications

The dual modification method is widely used for starch modification, as it can address the shortcomings of the single modification method. The dual modification method is developed using a combination of two modification methods. It can be broadly classified into six categories: physical–physical modifications, physical–chemical modifications, physical–biological modifications, chemical–chemical modifications, chemical–biological modifications, and biological modifications. There is a significant correlation between the physicochemical properties of dual-modified starch samples [133]. The dual modification methods have the potential to make starch widely available and, therefore, to expand the starch market. 

### 6.1. Physical–Physical Modifications

Dual physical modification was more effective than single physical modification. Zhou et al. compared the physicochemical properties of corn starch and potato starch modified by ultrasonic and microwave alone and by dual treatment [134]. The results showed that dual modification significantly affected the solubility, dehydration rate, and in vitro digestibility of potato starch. Colussi et al. investigated the relationship between humid HMT and HHP and evaluated their effects on thermal, pasting, swelling power, solubility, morphological and crystallization properties and in vitro digestibility of potato starch [135]. The results showed that when HHP was applied to HMT starch, the peak viscosity, retracted viscosity, and final viscosity were greatly increased compared to the samples treated with HMT alone. Dual modification increased the transition temperature and swelling force and changed the relative crystallinity. In addition, the dual-modified starch could be used as a substitute for low glycemic index foods. The results of this study are in agreement with Zhou et al. [134].

### 6.2. Physical–Chemical Modifications

The combined physical and chemical modification facilitates the further acquisition of starch with target properties. Li et al. compared the granular, physicochemical, and thermal properties of single-modified and corresponding freeze–thaw-assisted acid hydrolysis starches [136]. The study showed that after freeze–thaw and acid hydrolysis treatments, the starches exhibited the characteristic combination presented by both treatments alone. In addition, the oil adsorption capacity and solubility of the starches were significantly increased. The reasons may be the freeze–thawing exposes starch to more enzyme attachment sites, thus enhancing starch hydrolysis [137]. FITR and SEM showed that the chemical groups of the starches were not changed after the combined treatment, but Khurshida et al. revealed that the dual modification of cassava starch using ultrasound and acetic acid acetylation showed that the dual-modified starch had the lowest viscosity, and they pointed out that the type of dual modification and the order of modification had a significant effect on the physicochemical properties of the starch, and the solubility of the starch treated with ultrasound first was significantly lower than that of the starch treated with acetic acid acetylation first. Acetylation of starch was performed first [138]. Thus, the combination of physical and chemical modifications can help us to obtain starches with significantly different properties.

### 6.3. Physical–Enzyme Modifications

Physical modification methods are currently the preferred method for starch modification due to their green, efficient, and easy-to-operate nature, while enzymatic hydrolysis is used as a green modification method. Davoudi et al. investigated the effect of low-temperature plasma treatment and enzymatic hydrolysis on the physicochemical properties and microstructure of porous corn starch, and SEM images showed that deeper grooves appeared on the surface of the combined starch, and the combined starch showed the highest solubility, swelling capacity, and total pore volume [139]. Lin et al. investigated the structure and physicochemical properties of lotus seed starch nanoparticles prepared by ultrasound-assisted enzymatic hydrolysis, and the results showed that ultrasound further degraded the lotus seed starch nanoparticles [140]. However, when the ultrasound power and time were too large, some fine particles would adhere to the large particles so that the pullulanase could not fully function. At present, few studies have been reported for the control of the parameters of the dual modification method on the physicochemical properties of the modified starch; therefore, further studies are needed to better obtain products with the target properties.

### 6.4. Chemical–Chemical Modifications

Dual-chemical modification makes full use of the advantages of single-chemical modification, and therefore, its modification effect is more obvious. Javadian et al. investigated the effect of dual-chemical modifications on the functional, microstructural, and thermal properties of cassava starch by acid hydrolysis and then by hydroxypropylation of cassava starch [141]. The results showed that the solubility of the dual-modified starch was significantly higher than that of natural starch, that the pasting degree and pasting parameters of the dual-modified starch were lower, and that the dual modifications reduced the energy required to change the properties of the starch compared to single modifications. Sriprablom et al. compared the effects of cross-linking and octenyl succinylation mono- and bi-modification on the physicochemical, in vitro digestibility, and emulsification properties of cassava starch and showed the results in terms of physicochemical and emulsification properties [142]. This was mainly due to the effect of the functional groups introduced in the second modification, while in vitro digestibility seemed to be affected by the two functional groups introduced in the first and second modifications. This finding will help in the future acquisition of the target properties of starch.

### 6.5. Enzyme–Enzyme Modifications

The inherent advantages of the biomodification approach are: “green” and efficient. Dual biomodification further consolidates this advantage and effectively enhances that of single biomodification. Jeong et al. investigated the effect of α-amylase in combination with α-amylase and cellulase on the physicochemical properties of oat flakes and starch and showed that enzyme modification resulted in a significant increase in starch solubility and swelling and that the combined enzymes caused more intense depolymerization of starch molecules than single enzymes [143]. Guo et al. modified potato starch granules with branching enzymes and transglucosidase [144]. It was shown that during the enzymatic digestion of potato starch, the two enzymes exhibited synergistic effects, attacking the external and internal parts of the starch synergistically, and the complex enzyme modification increased the α-1,6-glycosidic bond ratio and the number of short chains of the starch compared to the single enzyme modification, resulting in a significant decrease in crystallinity, viscosity, pasting temperature and enthalpy, and in a significant increase in solubility. Table 5 summarizes the dual modification methods that have been reported for various starch sources.

## 7. Conclusions

Studies have shown that modified starch presents better physicochemical properties than native starch, and starch modification significantly expands the market for starch byproducts. Currently, people are placing great emphasis on eating well and healthily. With the rise in the concept of homology of medicine and food, the starch industry has been further boosted. For instance, Pueraria lobata starch high in isoflavones can be used as food and in the medicinal industry. As a result, it is a healthy and nutritious choice. Pueraria lobata starch is an underexplored native starch, and it will have great application in functional food ingredients if it is modified. Additionally, as the global population continues to grow, the development of modified starch with improved functionality and sustainability will become increasingly important to meet the demand for food and other industrial applications. Research on modified starch is expected to focus on developing more efficient and eco-friendly modification methods as well as on exploring novel starch sources and their potential uses in the future.

## Figures and Tables

**Table 1:** Summary of methods used for physical modifications of starch.

Type	Technique	Starch Source	Advantages	Influence on Structures and Properties	References
Thermal treatment	Hydrothermal treatment	Potato starch Wheat starch	Improve starch properties at low cost using a simple Environmentally friendly process	ANN and HMT: Both modifications increased tensile strength, elongation at break (%), reduced solubility and water vapor permeability. SS: The treated samples showed lower swelling power, solubility and viscosity, but had higher pasting temperature.	[13,22]
	Microwave	Sorghum starch	Efficient and green Easy to obtain equipment	Rough surface of starch granules. Increase in pasting temperature and pasting time. Chemical groups remain unchanged.	[27]
	Spray drying	Cassava starch	Resulting in retention of particle integrity Easy access to equipment	High cold viscosity. Low decomposition.	[31]
	Drum drying	Tapioca starch Maize starch	Economical Convenient	Increased tensile strength of bitter wheat flour flakes. Increased viscosity and water absorption index.	[36]
	Extrusion	Corn starch Potato starch	Versatile, efficient Economical production	Increased water absorption index, water solubility index, starch pasting. Decreased stability. Improved in vitro digestibility.	[39,46]
Non-thermal treatment	Ball milling	Wheat starch A-and B-type starch	Cost-effective Environmentally friendly	With increased solubility. Starch granule surface was disrupted. Increased amount of rapidly digestible starch.	[61]
	Ultrasonication	Cassava starch	Environmentally friendly	Degradation of starch polymer. Increased solubility.	[49]
	Radiation	Potato starch	Fast, efficient, non-toxic, chemical residue free Low heat production	Reduce molecular weight and viscosity. Improves starch solubility and swelling capacity.	[54]
	High hydrostatic pressure	Maize starches Potato starches Sweet potato starches lotus seed starch-fatty acid complexes	Easy to control Environmentally friendly	Increased content of amylose starch and damaged starch. Decreased solubility and swelling capacity. Improved crystallinity and stability.	[64,67]
	Supercritical CO_2_	Corn starches	Easy to operate Safe and non-toxic Low price	The introduction of different functional group substituents has different effects on their physicochemical properties.	[71]

**Table 2:** Summary of methods used for chemical modifications of starch.

Type	Technique	Starch Source	Advantages	Influence on Structures and Properties	References
Oxidation	Oxidation	Jackfruit seed starch	No harmful byproducts. Very environmentally friendly. High recovery rate.	Starch granules are rough, not smooth, with dents and cracks on the surface. Oxidation in the amorphous region of starch.	[80]
	Electrolyzed	Cassava starch	Safe and green. Effective in changing starch properties.	Rough surface of starch granules with large holes. Increased transparency. Increased solubility.	[77]
	Ozone-modified	Potato starch Buckwheat starch	Simple and environmentally friendly.	Higher enzyme sensitivity. Fully glued at lower temperature, easier to dissolve. Retains more water than natural starches.	[81,84,86]
Etherification	Hydroxypropylation	Wheat starch Waxy wheat starch Waxy maize starch	Inhibits the arrangement of starch chains. Significantly changes the properties of starch.	Decrease in pasting temperature and peak temperature. Increased hot and cold viscosity. Different degrees of starch swelling.	[90]
Esterification	Octenyl succinic anhydride	Japonica Rice Starches	Retains starch morphology and particle size, but starch properties change.	Reduced relative crystallinity and pasting temperature of starch. Improved pasting viscosity, swelling capacity, emulsification stability.	[105]
Acid hydrolysis	Acid hydrolysis	Achira starch	Can significantly change the structural and functional properties of starch without destroying the morphology of the starch granules.	Significant increase in solubility and water-holding capacity. Degradation of the amorphous and crystalline regions.	[115]

**Table 3:** Summary of methods used for enzymatic modifications of starch.

Type	Technique	Starch Source	Advantages	Influence on Structures and Properties	References
Enzymatic modification	Pullulanase and amyloglucosidase	Cassava starch	Green and Safe. Higher viscosity of pullulanase modified starch. Improves the shelf life of yogurt-based drinks.	Alters the water activity of yogurt-based beverages. Reduces lactic acid buildup over time. Higher viscosity of beverages with enzyme modified starch.	[122]
	α-amylase and glucoamylase	Cassava Starch	Easy access to target products. Green and safe.	Different enzymatic hydrolysis reactions for different sources of starch. Increase in apparent amylose content. Increased water binding capacity and water holding capacity.	[125]
	Glucoamylase and Ultrasound Assist	Potato starch	Accelerated enzyme hydrolysis.	Hydrogen bonds and starch chains are broken. Molecular weight reduction.	[126]

**Table 4:** Summary of methods used for genetic modifications of starch.

Type	Technique	Starch Source	Advantages	Influence on Structures and Properties	References
Genetic modification	CRISPR/Cas9	Sweet potato starch	Improve the quality of sweet potato starch. An effective tool for breeding polyploid tubers in crops. High mutagenesis rate.	In the allopolyploid sweet potato, the IbGBSSI-knockout reduced, while the IbSBEII-knockout increased, the amylose percentage.	[127]
		Tapioca starch	Efficient editing and wide versatility.	Increased content of resistant starch and amylose starch. Viscosity increases with pasting temperature and peak time.	[128]
		Rice starch	Production of high straight-chain starch.	Significant increase in resistant starch content.	[130]
		Rice starch	Produced four types of waxy rice starch with different physicochemical properties	Water solubility decreased with an increase in amylose content in waxy mutant starches. Decreased crystallinity. Different waxy mutant starches have different gel properties.	[131]

**Table 5:** Summary of methods used for dual modifications of starch.

Type	Technique	Starch Source	Advantages	Influence on Structures and Properties	References
Physical–physical modifications	Ultrasonic microwave	Corn starch, Potato starch	Dual modification is more effective than single modification.	Increased solubility of starch. Improved water and oil absorption capacity. Improved freeze-thaw stability.	[134]
	HMT-HHP	Potato starch	Can be used as a substitute for low glycemic index foods.	Increase in peak viscosity, retraction and final viscosity. Slower glucose release rate.	[135]
Physical–chemical modifications	Freeze–thaw acid hydrolysis	Corn starch	Freeze–thawing exposes starch to more enzyme attachment sites, thus enhancing starch hydrolysis [137].	Reduced amylose starch content. Increased solubility. Increased water and oil adsorption capacity. Decreased swelling capacity.	[136]
	Ultrasound acetylation	Tapioca starch	Modification order affects starch affects starch properties.	Decrease in swelling degree. Decrease in paste viscosity. Increase in resistant starch and slow-digesting starch content.	[138]
Physical–enzyme modifications	Ultrasound enzyme hydrolysis	Lotus Seed Starch	Ultrasound facilitates the rearrangement of starch amylose molecules.	Affected the starch size. Promoted molecular rearrangement during enzymatic hydrolysis.	[140]
Physical–enzyme modifications	Cold plasma enzymatic hydrolysis	Corn starch	Cold plasma promotes enzyme performance. Higher starch absorption capacity.	Increased swelling capacity and solubility. Decrease in the orderly arrangement of starch molecules. Delayed starch pasting.	[139]
Chemical–chemical modifications	Acid hydrolysis- etherification	Cassava starch	Reduces the force and energy required to modify starch.	Increase in solubility. Paste parameter and paste degree decrease.	[141]
	Cross-linking acetylation	Cassava starch	Expanding the range of applications.	Emulsification stability is improved. Thermal stability improvement.	[142]
Enzyme modifications	α-amylase enzyme cellulase	Oat Starch	Increased water absorption capacity of starch. Improved sensory evaluation of starch.	Swelling power, increased solubility. Degradation of polysaccharide chains.	[143]
	Branching enzyme transglucosidase	Potato starch	Ideal physical and chemical properties of potato starch can be obtained.	Crystallinity, viscosity and pasting temperature decrease. Increase in solubility.	[144]

## Data Availability

All basic data supporting the results of this study are available from the corresponding author.
